# Aerobic exercise improves executive functions in females, but not males, without the BDNF Val66Met polymorphism

**DOI:** 10.1186/s13293-023-00499-7

**Published:** 2023-04-03

**Authors:** Cindy K. Barha, Samantha Y. Starkey, G. Y. Robin Hsiung, Roger Tam, Teresa Liu-Ambrose

**Affiliations:** 1 Aging, Mobility, and Cognitive Neuroscience Lab, Department of Physical Therapy, University of British Columbia, Vancouver, Canada; 2 Djavad Mowafaghian Centre for Brain Health C/O Liu-Ambrose Lab, 2215 Wesbrook Mall, Vancouver, BC V6T 2B5 Canada; 3 Centre for Hip Health and Mobility, Vancouver, Canada; 4 Faculty of Medicine, University of British Columbia, Vancouver, Canada; 5 Division of Neurology, University of British Columbia, Vancouver, Canada; 6 Vancouver Coastal Health Research Institute and University of British Columbia Hospital Clinic for Alzheimer Disease and Related Disorders, Vancouver, Canada; 7 School of Biomedical Engineering, University of British Columbia, Vancouver, Canada; 8 Present Address: Faculty of Kinesiology, University of Calgary, Calgary, AB Canada

**Keywords:** Aerobic exercise, Executive function, Sex differences, Randomized controlled trial, BDNF Val66Met polymorphism, Vascular cognitive impairment

## Abstract

**Background:**

Aerobic exercise promotes cognitive function in older adults; however, variability exists in the degree of benefit. The brain-derived neurotropic factor (BDNF) Val66Met polymorphism and biological sex are biological factors that have been proposed as important modifiers of exercise efficacy. Therefore, we assessed whether the effect of aerobic exercise on executive functions was dependent on the BDNFval66met genotype and biological sex.

**Methods:**

We used data from a single-blind randomized controlled trial in older adults with subcortical ischemic vascular cognitive impairment (NCT01027858). Fifty-eight older adults were randomly assigned to either the 6 months, three times per week progressive aerobic training (AT) group or the usual care plus education control (CON) group. The secondary aim of the parent study included executive functions which were assessed with the Trail Making Test (B–A) and the Digit Symbol Substitution Test at baseline and trial completion at 6 months.

**Results:**

Analysis of covariance, controlling for baseline global cognition and baseline executive functions performance (Trail Making Test or Digit Symbol Substitution Test), tested the three-way interaction between experimental group (AT, CON), BDNFval66met genotype (Val/Val carrier, Met carrier), and biological sex (female, male). Significant three-way interactions were found for the Trail Making Test (F(1,48) = 4.412, *p* < 0.04) and Digit Symbol Substitution Test (F(1,47) = 10.833, *p* < 0.002). Posthoc analyses showed female Val/Val carriers benefited the most from 6 months of AT compared with CON for Trail Making Test and Digit Symbol Substitution Test performance. Compared with CON, AT did not improve Trail Making Test performance in male Val/Val carriers or Digit Symbol Substitution Test performance in female Met carriers.

**Conclusions:**

These results suggest that future randomized controlled trials should take into consideration BDNF genotype and biological sex to better understand the beneficial effects of AT on cognitive function in vascular cognitive impairment to maximize the beneficial effects of exercise and help establish exercise as medicine for cognitive health.

## Highlights

- Aerobic training improves cognitive health and identifying biological moderators of aerobic training will maximize the precision of exercise as medicine for cognitive health.
- The common BDNFval66met polymorphism and biological sex interact to moderate the cognitive-enhancing effects of aerobic training in older adults with vascular cognitive impairment, with female Val/Val carriers benefitting the most.
- Future randomized controlled trials of exercise should take into consideration genotype and biological sex of participants

## Background

Evidence supports aerobic training (AT) as a strategy to improve cognitive health, with the strongest evidence seen in older adults and for executive functions [1–3]. However, there is variability in the degree of benefit conferred by AT [2, 4] Biological sex and genotypic variation in key ageing-related genes have been proposed as important modifiers of AT efficacy [3, 5–8]. Increasing our understanding of biological moderators of the cognitive-enhancing effects of AT will maximize the benefits and help increase the precision of exercise as medicine for cognitive health.

It is hypothesized that one possible pathway through which AT benefits cognition is the induction of the brain-derived neurotrophic factor (BDNF) cascade, which supports neuroplasticity and the cellular mechanisms required for learning [9]. Rodent studies indicate that central BDNF levels mediate the beneficial effects of AT on the brain [10], with possibly greater effects in females [11], though currently is not known why this may be the case. Furthermore, meta-analytic evidence supports the role of BDNF in mediating AT efficacy in older humans [12].

Of relevance, a common functional single-nucleotide polymorphism exists within the pro-domain region of the BDNF gene resulting in an amino acid substitution of valine (Val) to methionine (Met) at position 66, termed the Val66Met substitution, and may influence exercise efficacy in a sex-dependent manner. The Met allele alters intracellular trafficking of the precursor form of BDNF, reducing the activity-dependent secretion of the mature form of BDNF [13]. Whether this BDNF polymorphism moderates the benefits of AT is not well-examined. To our knowledge, only one intervention study of AT has examined its role; the non-randomized trial showed AT improved global cognition regardless of BDNF genotype but only increased serum BDNF in Val/Val carriers [14].

No intervention study of AT has examined the interaction of the BDNFval66met polymorphism and biological sex on cognition. Findings from observational studies are equivocal. Sanders et al. [15] did not find an effect of the BDNFval66met genotype on the association between self-reported physical activity level and global cognition, regardless of sex. Using sex-stratified analyses, Watts et al. [16] found that self-reported physical activity level was associated with change in episodic and working memory in male Val/Val carriers but not Met carriers. Intriguingly, there are sex differences in the effects of the Met allele on hippocampal blood flow, age-related cognitive and brain volume decline and on Alzheimer’s disease risk [17–21].

Subcortical ischemic vascular cognitive impairment (SIVCI) is the most common form of vascular cognitive impairment in which covert ischemic strokes manifest as white matter hyperintensities, resulting in declines in executive functions, memory, language, gait disturbances, and increased falls [22]. Importantly, approximately 25% of adults over 80 years of age have had one or more silent brain infarcts, substantially increasing their risk of subsequent stroke and of vascular cognitive impairment [23]. In SIVCI, AT has been shown to improve general cognitive function, executive functions and neural activity [24, 25]. Interestingly, the beneficial effect of AT on executive functions is seen only in those, prior to study entry, were at lower cardiovascular risk [26]. Sex differences have also been found in the effect of AT on BDNF levels in people with SIVCI [27].

Therefore, we analyzed data collected from a single-blind randomized controlled trial (NCT01027858) to assess whether the effect of a 3x/week progressive AT intervention on executive functions and processing speed in participants with SIVIC [25] was moderated by the BDNFval66met polymorphism and biological sex.

## Methods

### Study design

The data for this analysis originated from a 26-week randomized controlled trial at the University of British Columbia campus at the Vancouver General Hospital. The primary findings and methods of this randomized controlled trial are published [25, 28]. Physical and cognitive assessments were conducted at baseline and trial completion at 6 months after randomization. All assessors were blinded to group allocation and participants were randomized 1:1 to AT or usual care plus education (CON). Ethics approval was obtained from the Clinical Research Ethics Board at the University of British Columbia (H07-01,160), and trial protocol was registered at ClinicalTrials.gov (NCT01027858) and published [28].

### Participants

All participants were recruited from the University of British Columbia Hospital Clinic for Alzheimer’s Disease and Related Disorders, the Vancouver General Hospital Stroke Prevention Clinic, and specialized geriatric clinics in Metro Vancouver, Canada. A complete description of the inclusion and exclusion criteria can be found in [25, 28]. Participants were clinically diagnosed with SIVCI by a neurologist and met the following three study criteria [29]: (1) the presence of both periventricular and deep white matter lesions and the absence of cortical or cortico-subcortical strokes or other causes of white matter lesions on clinical computer tomography or magnetic resonance images scans; (2) mild cognitive impairment, defined as a Montreal Cognitive Assessment score of < 26/30 [30]; and (3) Mini-Mental State Examination score of ≥ 20 [31]. Participants also had to be 55 years or older and functionally independent. Individuals were not eligible to participate if they were diagnosed with dementia of any type or another neurological condition as determined by a neurologist, or were taking medications that influenced cognitive function. All participants provided written informed consent.

### Descriptive and demographic variables

Baseline age, sex, education level, weight (kg), height (cm), body mass index (kg/m^2^), waist-to-hip ratio (waist circumference/hip circumference), and resting heart rate were collected. Montreal Cognitive Assessment and Mini-Mental State Examination assessed global cognition. Current physical health and functioning was assessed by: (1) Functional Comorbidity Index [32], (2) Short Physical Performance Battery [33], and (3) Timed Up-and-Go Test [34]. The 15-item Geriatric Depression Scale screened for depression [35]. The 6-Minute Walk Test assessed general functional fitness capacity [36]. The Physical Activity Scale for the Elderly assessed current physical activity [37].

### Executive functions

We previously found a sex-dependent effect of AT on the executive function of set-shifting using the Trail Making test in SIVCI adults [27]. Thus, the primary outcome in the current analysis was the Trail Making test (Part B minus Part A). Part A assesses psychomotor speed by requiring participants to draw lines between encircled numbers connecting them in ascending order as quickly and as accurately as possible. Part B requires participants to connect alternating numbers and letters in ascending order. The amount of time (in seconds) to complete each part was recorded. Set-shifting was indexed by calculating the difference in completion time between Parts B and A, with smaller difference scores indicating better performance.

Biological sex interacts with the BDNFval66met polymorphism to influence Digit Symbol Substitution Test [21], a measure of sustained attention, working memory and processing speed. Thus, the secondary outcome in the current analysis was the Digit Symbol Substitution Test. The test consists of 9 digit-symbol pairs. Participants are required to fill in as many corresponding symbols for the given digits within 90 s. The total number of items correctly coded was scored, with higher scores indicating better performance.

### Experimental groups

#### Aerobic Training group (AT)

Classes were led by certified fitness instructors, were 60-min in duration (10-min warm-up, 40-min walking, 10-min cool down), and occurred 3×/week for 6 months. Adherence was monitored by recording class attendance. The AT intensity was monitored via heart rate monitors as well as the 20-point Borg Rating of Perceived Exertion [38]. Intensity was initially set at 40% of each participant’s heart rate reserve and Rating of Perceived Exertion of 11. Participants were progressed slowly over the first 12 weeks to reach a target of 60–70% of heart rate reserve and once 65% heart rate reserve was achieved it was maintained for the rest of the trial. Rating of Perceived Exertion was progressed to a target of 14–15. In addition, the “talk” test was used, starting at a walking pace that allowed participants to converse comfortably and slowly progressed to a walking pace where conversation was hard to maintain.

#### Usual care plus education group (CON)

Participants in the CON group attended monthly education classes given by a registered dietician and read materials pertaining to vascular cognitive impairment and healthy diets. Information regarding exercise and physical activity were not provided.

### Genotyping

Peripheral whole blood samples were collected in BD Vacutainer lavender top blood collection tubes at baseline and were stored at − 80 °C. DNA was extracted from whole blood using an automated DNA extraction machine (AutoGen FLEX STAR, Hollisten, MA). Genotyping of the BDNFval66met polymorphism (GenBank dbSNP: rs6265) was performed with TaqMan SNP assay (C-11592758-10) in an optimized ABI 7300 (Applied Biosystems). Due to the low frequency of the Met/Met genotype in the population (less than 5%), the established convention in the field was followed and Met/Met and Val/Met participants were combined into a single group.

### Statistical analyses

The statistical package SPSS 23.0 (IBM Corporation Armonk, NY) was used to conduct analysis of covariance to evaluate the main effects of and interactions between experimental group (AT, CON), BDNF genotype (Val/Val carrier, Met carrier), and biological sex (female, male) for the primary (i.e., Trail Making Test) and secondary (i.e., Digit Symbol Substitution Test) outcomes. Baseline Montreal Cognitive Assessment score and baseline score for the outcome variable were entered as covariates in all analysis of covariance models. Analyses were conducted as complete case analyses. Where there was a significant interaction effect, posthoc analyses were then performed using a Bonferroni correction, accounting for the overall correlation between the two measures of executive functions, the Trail Making Test and Digit Symbol Substitution Test. To interpret the three-way interaction of experimental group*BDNF genotype*biological sex, we conducted posthoc comparisons between AT and CON groups in male and female Met carriers and Val/Val carriers separately. Effect sizes for significant results were calculated as partial eta squared (η_p_^2^).

## Results

### Sample description

**Fig. 1:**
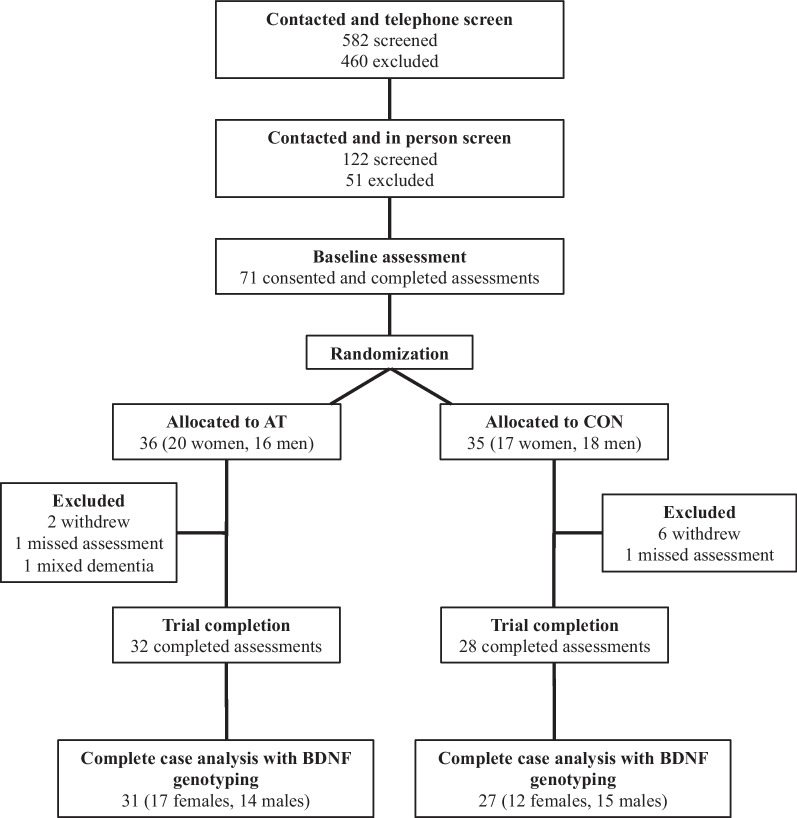
CONSORT diagram for the flow of participants in a randomized controlled trial

Of the 71 participants enrolled in the original trial, the present analyses are based on 58 of those participants that completed the trial with BDNF genotyping (Fig. 1). Table 1 provides the sample size and the baseline characteristics by experimental group, BDNF genotype, and biological sex. Groups did not differ in any of the baseline characteristics except baseline weight [F(1,50) = 4.793, *p* < 0.034]. Closer inspection showed that CON and AT groups only significantly differed on baseline weight in female BDNF Val/Val carriers (*p* < 0.048). Genotype distribution in the sample was 66% Val/Val, 29% Val/Met, and 5% Met/Met, and this distribution fit the Hardy–Weinberg equilibrium (χ^2^ = 0.35 *p* = 0.84). This subsample did not significantly differ from the parent sample on baseline characteristics (see Table 2).

**Table 1:** Baseline characteristics of male and female BDNF Val/Val carriers and Met carriers in the two experimental groups, mean (standard deviation)

BDNF genotype	Usual care plus education group				Aerobic training group				*p* value
	Males		Females		Males		Females		
	Val/Val	Met	Val/Val	Met	Val/Val	Met	Val/Val	Met	
Descriptive variables									
n	8	7	6	6	10	4	14	3	
Age, year	74.4 (9.9)	76.3 (8.7)	70.3 (4.3)	74.2 (4.9)	76.6 (9.0)	68.3 (8.1)	72.9 (6.9)	75.7 (7.2)	0.316
Functional comorbidity index	4.1 (2.9)	3.3 (1.6)	3.8 (2.6)	4.0 (2.6)	3.6 (1.3)	1.5 (1.3)	3.5 (1.3)	3.0 (2.6)	0.804
Waist to hip ratio	0.96 (0.03)	0.95 (0.04)	0.86 (0.08)	0.91 (0.08)	0.94 (0.07)	0.90 (0.05)	0.83 (0.06)	0.82 (0.10)	0.798
Body Mass Index, kg/m^2^	26.8 (2.1)	27.3 (3.0)	27.4 (2.9)	24.7 (5.1)	26.7 (3.7)	25.8 (5.2)	24.6 (3.4)	25.5 (2.8)	0.223
Weight (kg)	81.7 (7.0)	81.6 (9.4)	69.7 (7.6)	57.9 (13.0)	83.8 (10.5)	80.1 (13.5)	60.0 (8.9)	69.4 (10.2)	0.033
Resting heart rate, bpm	71.3 (19.1)	62.7 (14.7)	73.0 (18.7)	75.3 (11.6)	67.2 (14.6)	63.0 (20.5)	67.6 (9.8)	70.3 (14.2)	0.820
Short physical performance battery	10.3 (0.9)	10.3 (1.7)	10.7 (0.8)	11.2 (0.8)	10.9 (1.3)	12.0 (0.0)	10.5 (1.8)	11.7 (0.6)	0.791
Timed Up-and-Go Test, sec	8.4 (1.9)	9.6 (4.1)	8.1 (1.3)	7.6 (1.5)	8.8 (1.9)	7.7 (1.3)	8.4 (2.3)	7.5 (1.8)	0.491
Physical activity scale for the elderly	122.9 (62.4)	118.3 (59.9)	145.9 (40.9)	130.4 (59.1)	125.9 (110.9)	137.1 (22.6)	133.8 (65.2)	85.6 (26.8)	0.553
6-Minute Walk Test, m	461.6 (96.3)	480.0 (105.9)	528.0 (79.7)	516.5 (109.9)	520.1 (87.7)	575.8 (92.0)	488.0 (87.9)	578.0 (98.1)	0.559
Geriatric Depression Scale	2.9 (2.0)	2.4 (2.5)	1.67 (1.9)	1.67 (1.4)	2.2 (2.8)	1.0 (2.0)	2.3 (2.4)	2.0 (1.7)	0.860
Cognitive variables									
Mini-Mental State Examination	26.5 (2.9)	26.4 (2.9)	28.0 (1.4)	27.7 (1.6)	25.4 (3.0)	26.8 (1.7)	27.1 (2.7)	25.7 (3.1)	0.414
Montreal Cognitive Assessment	23.0 (2.6)	22.1 (3.8)	23.2 (2.5)	22.7 (3.1)	21.1 (3.9)	22.5 (0.6)	20.6 (2.6)	19.7 (4.0)	0.447
Trail Making Test B–A, sec	93.9 (117.9)	58.0 (18.0)	56.7 (38.3)	55.7 (27.9)	58.8 (65.8)	55.4 (15.6)	59.4 (31.1)	23.3 (6.9)	0.315
Digit Symbol Substitution Test	23.4 (3.5)	24.1 (4.0)	29.5 (9.4)	23.5 (7.6)	20.8 (5.3)	25.3 (9.8)	28.4 (7.8)	29.3 (7.6)	0.689

**Table 2:** Baseline characteristics of participants in the parent (full) sample and the complete case sample, mean (standard deviation)

	Parent sample	Complete case	*p* value
Age, year	73.9 (7.9)	73.8 (1.0)	0.949
Functional comorbidity index	3.5 (2.1)	3.5 (1.9)	0.912
Waist to hip ratio	0.9 (0.1)	0.9 (0.1)	0.856
Body Mass Index, kg/m^2^	26.2 (4.9)	26.0 (3.5)	0.829
Weight (kg)	71.2 (14.3)	72.4 (13.9)	0.651
Resting heart rate, bpm	68.6 (13.8)	68.6 (14.5)	0.981
Short physical performance battery	10.7 (1.5)	10.8 (1.3)	0.732
Timed Up-and-Go Test, sec	8.5 (2.3)	8.4 (2.2)	0.807
Physical activity scale for the elderly	125.2 (64.9)	127.7 (66.6)	0.831
6-Minute Walk Test, m	500.4 (95.6)	506.7 (94.0)	0.714
Geriatric Depression Scale	2.3 (2.4)	2.1 (2.2)	0.611
Cognitive variables			
Mini-Mental State Examination	26.3 (2.9)	26.7 (2.6)	0.429
Montreal Cognitive Assessment	21.2 (3.9)	21.8 (3.1)	0.351
Trail Making Test B–A, sec	67.3 (67.2)	61.1 (55.6)	0.580
Digit Symbol Substitution Test	24.9 (7.5)	25.3 (7.2)	0.726

### Exercise adherence and physical activity levels outside of the study

Adherence to AT did not differ between male and female Val/Val and Met carriers [BDNF genotype x sex interaction: F(1,29) = 1.817, *p* > 0.05, η_p_^2^ = 0.059]. At trial completion, the 6-Minute Walk Test and Physical Activity Scale for the Elderly did not differ between male and female Val/Val and Met carriers in the CON and AT groups (all main and interaction effects *p*’s > 0.088; see Table 3).

**Table 3:** Estimated adjusted within-group mean change (standard error) from baseline to trial completion in performance on tests of executive functions, functional fitness capacity and physical activity

	Usual care plus education group				Aerobic training group			
	Males		Females		Males		Females	
	Val/Val	Met	Val/Val	Met	Val/Val	Met	Val/Val	Met
*n*	8	7	6	6	10	4	14	3
Trail Making Tests B–A, sec*	− 3.14 (20.7)	5.16 (20.7)	46.11 (22.6)	3.94 (22.4)	57.50 (17.4)	− 2.68 (27.4)	− 28.01 (15.0)	− 0.86 (33.2)
Digit Symbol Substitution Test, total score*	0.82 (1.1)	1.63 (1.2)	2.29 (1.4)	− 3.12 (1.3)	1.35 (1.0)	− 0.92 (1.5)	− 1.48 (0.9)	2.02 (1.9)
6-Minute Walk Test, m	− 4.87 (19.0)	− 11.53 (18.5)	− 22.59 (19.9)	28.31 (19.8)	53.39 (16.3)	38.64 (24.5)	6.91 (13.7)	− 0.68 (29.1)
Physical Activity Scale for the Elderly	− 34.54 (22.5)	− 13.76 (22.2)	− 31.55 (27.6)	− 18.37 (27.3)	12.01 (21.1)	− 8.59 (29.9)	10.20 (18.0)	20.43 (34.1)

Change scores are adjusted for baseline Montreal Cognitive Assessment score and baseline score

For all tests of executive functions, a negative change score indicates an improvement over time. For the 6-Minute Walk Test and Physical Activity Scale for the Elderly, a negative change score indicates a decrease in performance over time

^*^indicates significant sex x BDNF genotype x experimental group effect, *p* < 0.05

### Sex and genotype dependent effect of aerobic training on executive functions

Table 3 presents the change in performance on the primary outcome variable, the Trail Making Test, and the secondary outcome, the Digit Symbol Substitution Test, adjusted for covariates baseline Montreal Cognitive Assessment score and baseline executive test score, from baseline to trial completion for male and female participants by experimental group and BDNF genotype.

**Fig. 2:**
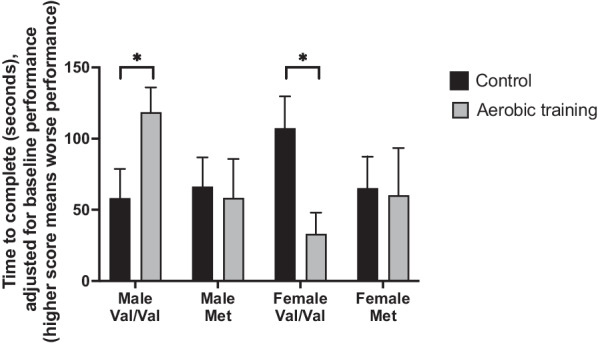
Total time taken to complete the Trail Making Test (B–A) in seconds at trial completion, adjusted for baseline Montreal Cognitive Assessment and baseline Trail Making Test (B–A) scores. A 6-month aerobic training program significantly improved performance in female brain-derived neurotrophic factor (BDNF) Val/Val carriers and decreased performance in male BDNF Val/Val carriers compared with a usual care plus education control program. No difference was seen in female and male Met carriers

There was a significant three-way interaction between experimental group, BDNF genotype, and sex [F(1,48) = 4.412, *p* < 0.041, η_p_^2^ = 0.084; see Fig. 2] for Trail Making Test performance at trial completion, with AT significantly improving Trail Making Test performance in female Val/Val carriers compared with female Val/Val CON (*p* < 0.010). In contrast, AT-trained male Val/Val carriers demonstrated significantly reduced Trail Making Test performance compared with CON male Val/Val carriers at trial completion (*p* < 0.033). Among Met carriers, AT did not influence Trail Making Test performance compared with CON females or males (*p* > 0.802).

**Fig. 3:**
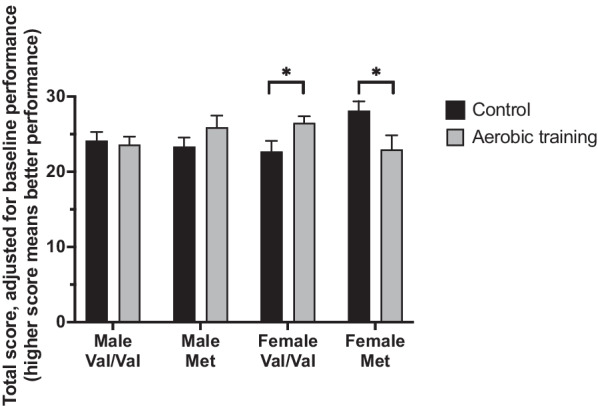
Total score on the Digit Symbol Substitution Test at trial completion, adjusted for baseline Montreal Cognitive Assessment and baseline Digit Symbol Substitution Test scores. A 6-month aerobic training program significantly improved performance in female brain-derived neurotrophic factor (BDNF) Val/Val carriers and decreased performance in female BDNF Met carriers. No difference was seen in male Val/Val or Met carriers

A significant three-way interaction was observed between experimental group, BDNF genotype, and sex [F(1,47) = 10.833, *p* < 0.002, η_p_^2^ = 0.187; see Fig. 3] for Digit Symbol Substitution Test performance, with AT significantly improving performance in female Val/Val carriers compared with female Val/Val CON (p < 0.027). In contrast, AT-trained female Met carriers demonstrated significantly reduced Digit Symbol Substitution Test performance compared with CON female Met carriers (p < 0.032). Among males, AT did not influence Digit Symbol Substitution Test performance compared to CON in Met or Val/Val carriers (p > 0.190).

Overall, analyses indicate that 6 months of AT increases performance on two tests of executive functions compared to CON in female BDNF Val/Val carriers (see Figs. 2 and 3).

## Discussion

Results from this randomized controlled trial provide preliminary evidence that BDNFval66met genotype interacts with biological sex to influence AT efficacy on executive functions in older adults with SIVCI, a clinical population with increased dementia risk. Specifically, we found that engaging in 6 months of AT compared with usual care and education control led to improvements on the Trail Making test (set-shifting) and Digit Symbol Substitution Test (working memory, sustained attention, and processing speed) in female Val/Val carriers. AT decreased performance on the set-shifting test in male Val/Val carriers and decreased performance on the working memory, sustained attention and processing speed test in female Met carriers compared with control.

While the evidence for engaging in exercise to promote cognition is mounting [1, 2], variability in the degree of benefit still exists [2, 4]. It has been postulated that this variability in outcomes may be related to moderation of exercise efficacy by genotype and sex of participants [5–8]. The findings from the present study provide further evidence supporting the importance of sex of participants as a source of variability requiring attention in exercise trials as has been previously shown [3, 39–41], and further suggests that sex interacts with the BDNFval66met polymorphism. Previous work examining this polymorphism have mainly been done in the context of observational studies, with equivocal results as the positive association between physical activity and cognition was seen only in Met carriers, only in Val/Val carriers or not seen in either group [42], with the majority of studies in cognitively unimpaired older adults. Kim et al., [43], on the other hand, examined the relationship between physical activity and global cognitive function in other adults with prevalent dementia. They found in older adults with lower physical activity, Met carriers had the most impaired global cognition, but in those with high physical activity no difference was seen between Val/Val and Met carriers. In a non-randomized 2-arm study, Nascimento et al. [14] examined the effect of a 16-week multimodal intervention compared with sedentary control on performance on the Montreal Cognitive Assessment, a screening tool for cognitive impairment, in older males and females with mild cognitive impairment and found no difference as a function of genotype. Our current findings indicate that BDNF genotype moderates AT effects on executive functions in those with SIVCI. Thus, it may be the case that BDNF genotype effects may be more specific to aerobic exercise compared to other forms of exercise that were included in the multimodal intervention and are specific to the cognitive domain of executive functions, which also typically shows the greatest effect sizes associated with AT [3]. In addition, the clinical diagnosis of the participants under examination may be important for the moderating effect of BDNF genotype, with SIVCI showing the effect. We do not believe the differences in our findings from those of Nascimento et al. [14] are related to the differences in the length of the exercise interventions (6 months vs. 16 weeks) as a previous meta-analysis found similar effect sizes of exercise training across intervention lengths (4 weeks to greater than 26 weeks) [44]. In addition, our results suggest that biological sex is an important effect modifier that should be considered in relation to the BDNF polymorphism. Importantly, we found that BDNF genotype was more influential on exercise efficacy in females, with female Val/Val carriers showing beneficial effects of AT on executive functioning and female Met carriers not showing the same beneficial effects. In contrast, in an observational study, Watts et al. [16] found that the association between engagement in physical activity and memory was only influenced by BDNF genotype in males, with moderate and vigorous physical activity associated with slower declines in memory compared with low or no physical activity in Val/Val carriers. The apparent discrepancies between these two studies could be related to the cognitive domains being studied (executive functions vs. memory), study design (randomized controlled trial vs. observational), or exercise vs. physical activity. Taken together, these findings support the need for future studies of exercise to consider biological sex, the BDNFval66met polymorphism and their interaction as effect modifiers.

Our findings regarding the effect of AT on executive functions in Val/Val carriers are in line with prior observational studies that found the association between physical activity and executive functions was mainly evident in Val/Val carriers, though biological sex was not examined in these studies. For example, on tests of the executive function of conflict resolution, Val/Val carriers that were physically active performed better than inactive Val/Val carriers [45] as did Val/Val carriers with higher self-reported levels of physical activity [46]. Furthermore, Erickson et al. [47] found that Val/Val carriers with lower physical activity had better working memory than Met carriers with lower physical activity, and similar levels of performance was seen in Val/Val and Met carriers with higher physical activity. We extend these findings to show that the stronger relationship between physical activity and exercise in Val/Val carriers may be most evident in females.

As outlined by de las Heras and colleagues [42], there are two main hypotheses for how the BDNF polymorphism may be exerting its influence on exercise efficacy for cognition. Specifically, Met carriers may have greater cognitive gains from AT due to their lower baseline levels of activity-dependent secretion of BDNF. Our findings do not support this hypothesis and actually indicate that AT is ineffective in increasing cognition in male and female Met carriers. Alternatively, AT may be more effective in improving cognition in Val/Val carriers as they have greater activity-dependent secretion of BDNF compared with Met carriers, thus AT may induce higher levels of BDNF within the brain. Indeed, Nascimento et al. [14] using a non-randomized two-arm study design, found that a multimodal exercise intervention that included an AT component increased peripheral circulating levels of BDNF in only Val/Val carriers. Our finding that AT improved executive functions in only female Val/Val carriers, taken together with our previous finding that AT only increased BDNF levels in females [27], further provides support for this hypothesis with the caveat that it may be more relevant for females.

Previous work suggests that the effect of BDNFval66met polymorphism on hippocampal blood flow, age-related cognitive and brain volume decline, and Alzheimer’s disease risk is sex-dependent [17–21]. This list now includes AT effects on executive functioning. Previous work suggests that the Met allele confers increased risk for Alzheimer’s disease in females but not in males [17], though this association is not always found [48] Female Met carriers have also been shown to have lower performance on the Digit Symbol Substitution Test than female non-carriers [18]. In line with this, our study provides preliminary evidence that AT may be detrimental to performance on the Digit Symbol Substitution Test in female Met carriers. The detrimental effect of the Met allele in females may be related to lower central BDNF levels in the brain as seen in a recent rodent study that found lower BDNF protein content in the ventral hippocampus in only female Met carriers compared with female Val/Val carriers [49]. In the periphery, the relationship between BDNF genotype and sex is still unclear; however, previous work did find that male Met carriers had higher serum BDNF levels compared with male Val/Val carriers, with no genotype effect in females [50]. Previous work has shown that in participants aged 18 to 50, resting regional cerebral blood flow was higher in female Val/Val carriers than male Val/Val carriers but in Met carriers it was higher in males than females [20]. This pattern of blood flow in female Val/Val and Met carriers may be dependent on estradiol levels as a study in similarly aged females (18–50 years) found that resting regional cerebral blood flow was higher in Met carriers than Val/Val carriers but only when estradiol levels were high [51]. Importantly, moderate-intensity AT can increase cerebral blood flow [52] in those with amnestic mild cognitive impairment. Whether AT during the perimenopause period when estradiol levels are changing can increase cerebral blood flow in a BDNF Val66Met dependent manner is not known.

## Limitations

As is the case with most studies examining the effects of polymorphisms on cognition, our analyses are limited by the small number of male and female BDNF Met allele carriers. The Met allele is found in 30–70% of the population, depending on ethnicity [53]. Participants were not genotyped for this polymorphism prior to randomization into the AT or control groups. However, by chance the distribution of the Met allele within these two groups was fairly equal. We did not correct our overall alpha but we focused on two specific measures of executive functions based on our prior findings [21, 27]. The consistent findings for the AT-trained female Val/Val carriers across these two tests support the hypothesis that sex interacts with the BDNF polymorphism to influence AT efficacy. However, despite the robustness of our findings, we are cautious in our interpretations and suggest that future studies that stratify randomization based on sex and genotype are warranted and should be conducted in other populations as our results may not be generalizable beyond those with SIVCI. In addition, we were not able to assess menopause status in our female participants, which could have an effect on our results. However, our female participants were between the ages of 59 and 84, and the median age of natural menopause in Canada is 51 years [54].

## Perspectives and significance

Dementia is one of the most pressing health care issues worldwide and vascular cognitive impairment is the second most common type of dementia. As an effective pharmacological treatment of dementia remains elusive, aerobic exercise has been identified as a promising strategy for preventing dementia and reducing key cardiometabolic risk factors for vascular cognitive impairment. To increase the utility and effectiveness of exercise, it is imperative to identify factors that moderate its cognitive-enhancing ability. In the present study, it was shown that biological sex and BDNFval66met polymorphism interact to moderate the effect of a 6-month AT program on executive functions and processing speed in older adults with SIVCI. Future large-scale randomized controlled trials of exercise on cognitive health should take into consideration sex and genotype to maximize the beneficial effects of exercise and help establish exercise as medicine for cognitive health. Our results indicate that to optimize the benefits of exercise, strategies should be tailored and personalized. For example, our results suggest that AT may be more beneficial for female BDNF Val/Val carriers with executive dysfunction. Intriguingly, patients with Frontotemporal Dementia show impaired executive functions [55], which may be greater in female patients than male patients [56] despite similar disease prevalence in males and females [57]. Thus, exercise strategies should be tailored and personalized based on variables such as biological sex, genotype and cognitive domain showing impairment [6, 7].

## Data Availability

The data sets used and analysed during the current study are available from the corresponding author on reasonable request.
